# CD4+ cells recovery in HIV positive patients with severe immunosuppression at HAART initiation at Centre Medico-Social Cor-Unum, Kigali

**DOI:** 10.11604/pamj.2017.26.14.10488

**Published:** 2017-01-12

**Authors:** Nyiramana Marie Merci, Uwimana Emerence, Nzitakera Augustin, Michael Habtu, Ingabire Julie, Tuyishime Angelique, Beneyo Jessica, Akimana Cynthia, Augustin Twizerimana Penda

**Affiliations:** 1 Mount Kenya University, College of Health Sciences, Department of Medical Laboratory Sciences, Kenya; 2 University of Rwanda, College of Medicine and health Sciences, Department of Biomedical Laboratory Sciences, Kigali, Rwanda; 3 Institute of Tropical Medicine and Infectious Diseases, Jomo Kenyatta University of Agriculture and Technology, Nairobi, Kenya

**Keywords:** HAART, CD4+ cells, viremia, severe immunosuppression, immune recovery

## Abstract

**Introduction:**

Up to 30% of HIV infected patients who are receiving HAART do not exhibit a marked increase in the CD4+ T cell count. There is still a concern that immune recovery may not be complete once CD4+ T cells have decreased below 200 cells/μl. The objective is to assess CD4+ cell recovery in HIV+ patients with CD4 count below 200 cells/μl) at HAART initiation.

**Methods:**

This was a retrospective cohort study among 110 HIV+ patients with initial CD4 count < 200 cells/μl. Baseline Age, sex, CD4 count and viral load were extracted from the patient’s database. After12 months of HAART; CD4 count was done using flow cytometry and viremia by COBAS AmpliPrep/COBAS TaqMan HIV-1 test v 2.0 technology.

**Results:**

The mean age of the respondents was 35 years; males being 57% and females were 43%. The mean CD4 count before HAART was 110.18 cells/μl whereas at 12 months of HAART; this was 305.01 cells/μl. Though some patients did not achieve a CD4 count of more than 200 cells/μl or a drop in viral load; there was a significant recovery of CD4+ cells (P value=0.000) and viremia following HAART (P value=0.001). Participants aged 18-30 years were likely to have less than 200 cells/μl CD4 count (46.4%) [OR=4.33; 95%CI: 1.29-14.59; P=0.018] than participants aged above 40 years (16.7%).

**Conclusion:**

HAART was associated with viremia suppression but many patients failed to achieve a CD4 count >200 cells/μl. HAART before severe immunosuppression is a key factor for immune restoration among HIV+ patients.

## Introduction

Human immunodeficiency virus (HIV) is a lentivirus; a member of the retrovirus family that causes Acquired Immunodeficiency Syndrome (AIDS) [1]. Human Immunodeficiency Virus Infection/Acquired Immunodeficiency Syndrome (HIV/AIDS) is among the most serious public health challenges man has ever faced; particularly in developing countries with low per capita income [2]. Approximately 35.0 million people were living with HIV worldwide by the end of 2013 [3]. The number of people receiving HIV treatment has tripled in five years and reached 9.7 million in low- and middle-income countries in 2012, and this represents 65% of the global target of 15 million people [4].

Work undertaken by the National Institute of statistics of Rwanda (NISR), in the 2010 RDHS, indicated that in Rwanda, HIV prevalence was 3.7% and 2.2% among women and men, respectively. The city of Kigali, Capital of Rwanda had the highest prevalence (7.3%) in the country [5]. Periodic CD4 count (every three months) and viral load (every 12 months) are used for HIV^+^ patients biological follow up that aims at assessing the infection progression as well as response to HAART [6]. The best limit CD4 count for HAART initiation is 350 cells/μl [7]. Previous studies have showed that factors including age, specific drug regimen, and initial CD4 count influence CD4 count recovery among patients with virological suppression [8–10]. A low CD4 count before treatment is a risk factor for not achieving a CD4 count > 200 cells/μl following HAART [11] and studies have documented that the lower the CD4 count at HAART initiation; the longer and more difficult it will be to achieve a net CD4+ recovery [12]. HIV+ are said to be undergoing a severe immunosuppression when their CD4 counts are below 200 cells/μl [13].

CD4 count has been reported to be one of the best surrogate markers for monitoring the progression of HIV infection and low CD4 counts are associated with increased risk of developing AIDS or death [14]. The absolute CD4 cell counts form the basis for antiretroviral therapy initiation and monitoring among HIV-infected adults though the rate of CD4 cell change differs among patients [7]. Mortality is among the late antiretroviral treatment outcome variables that have been studied extensively, and rates of up to 30% have been reported during the first year of treatment in some sub-Saharan settings [15]. Researches have documented a fast CD4+ cell recovery among patients with baseline CD4 count ≥ 200 cells/μl compared to patients with baseline CD4 <200 cells/μl [7, 16]. The present study aimed at assessing CD4+ cells recovery in HIV+ patients with severe immunosuppression (CD4 count <200 cells/μl) at HAART initiation at Centre Medico-Social Cor-Unum located in Kigali.

## Methods

This was a retrospective cohort study that started from March 2015 to February 2016. The study aimed at looking at how CD4+ cells recover when HIV+ patients initiate HAART with a severe immunosuppression. Baseline data including demographic (age and sex), CD4 count, and viral load were collected from HIV+ patient’s database (clinical file). The inclusion criteria were HIV+ patients who had initiated HAART with a CD4 count of less than 200 cells/μl.

The study was conducted at Centre Medico-Social Cor-Unum located in Kigali; the capital city of Rwanda. This health facility is a member of public health facilities in Rwanda that host voluntary counseling and testing (VCT), antiretroviral treatment services and follow up for many HIV+ patients. In addition, the area is near the National Reference Laboratory of Rwanda where viral load was to be measured and this has facilitated easy and cheap sample transportation at 12 months of HAART. Blood samples were collected from 110 study participants who had been on HAART for 12 months. The CD4 count was done using flow cytometry (BD FACSCount™ system) while viral load was determined using COBAS AmpliPrep/COBAS TaqMan HIV-1 test v2.0 technology.

Paired and independent t test were used to compare means between baseline CD4 cell; viremia and at 12 months of HAART initiation; as well as between demographics and CD4 cell/viral load respectively. Moreover, chi square test was used to determine the association between demographics and CD4 count level. The significance level was set at P value <0.005. This study was reviewed and approved by Mount Kenya University (MKU04/DPA/16/2015/3007) and Congregations Around Richmond Involved to Assure Shelter (CARITAS)-Rwanda as Centre Medico-Social Cor-Unum operates under CARITAS in partnership with the Rwanda Ministry of Health. All the patients signed the informed consent before participation to the study.

## Results

### Demographic characteristics of study participants

Table 1 presents the age and sex distribution of study participants. The mean age was 35 years and about half (47.3%) of the respondents were aged between 31-40 years. More males (57%) than females (43%) participated to the study.

**Table 1:** Demographic attributes of study participants

Variable	n=110	%
**Age in years**		
18-30	28	25.5
31-40	52	47.3
> 40	30	27.3
Mean age (+SD) = 35.2(+8.5)		
**Sex**		
Female	47	42.7
Male	63	57.3

### CD4+ cells count at HAART initiation and at 12 months of treatment

As shown in Table 2, the 110 HIV+ patients who participated to the present study had a mean CD4 count of 110.18 cells/μl at HAART initiation with a minimum CD4 count of 1 cell/μl and a maximum of 199 cells/μl. Following 12 months of HAART, the mean CD4 count raised to 305 cells/μl. The minimum and maximum CD4 count were 2 cells/μl and 1171 cells/μl respectively. Paired samples t test was computed to determine the mean difference (110.18 cells/μl before HAART and 305.01 cells/μl after 12 months of HAART). There was a significant recovery of CD4 positive cells following HAART among study participants (P value = 0.000).

**Table 2:** CD4^+^ cells count before HAART initiation and at 12 months of treatment

	Before HAART	At 12 months of HAART	Paired *t* test
Number of Patients	110	110	0.000
Mean	110.18	305.01	
Minimum CD4 count	1	2	
Maximum CD4 count	199	1171	

### CD4 count stratified by sex and age

Table 3 presents the levels of CD4+ cells based on sex and age. Before HAART initiation men had a mean CD4+ count of 109.57 cells/μl and for females the mean was 111 cells/μl. The difference was not significant (P value =0.899). Following 12 months of HAART, the mean CD4+ count among females was higher (311.81 cells/μl) compared to males (299.9 cells/μl). However, this difference was not significant (P value= 0.726). Similarly, there was no difference in CD4+ count between age groups before HAART initiation (P value= 0.536) as well as after 12 months of HAART (P value = 0.584).

**Table 3:** CD4 count based on gender and age. Mean CD4 counts among females and males and their comparison are presented

Variables	N	Mean	Std. Deviation	Independent *t* test/ANOVA test
**CD4+ cells Count before HAART initiation based on sex**				
Male	63	109.57	56.76	0.899
Female	47	111	60.16	
**CD4 count at 12 months of treatment based on sex**				
Male	63	299.94	152.98	0.726
Female	47	311.81	201.75	
**CD4+ cells Count before HAART initiation based on age**				
18-30 years	28	100.36	63.643	0.536
31-40 years	52	115.62	56.166	
> 40 years	30	109.93	56.251	
**CD4 count at 12 months of treatment based on age**				
18-30 years	28	297.14	248.405	0.584
31-40 years	52	322.44	147.725	
> 40 years	30	282.13	133.246	

### Viral load level among study participants

Viral load median was computed at both time points as shown in Table 4. Median viral load was 23400 HIV RNA copies/ml before HAART. When viral load was expressed in logs for a normal distribution, viral load median was equivalent to 4.3 logs before HAART initiation. After 12 months of HAART; the median viral load dropped to 20 HIV RNA copies/ml (equivalent to 1.3 logs) and the difference in viral load was significant between the two time points (P value = 0.000).

**Table 4:** Wilcoxon signed ranks test is used to highlight the difference in viral load between the two time points under this study

	Before HAART	At 12 months of HAART	Wilcoxon Signed Ranks Test
Number of Patients	110	110	0.000
Median (Logs)	23400(4.3)	20(1.3)	
Minimum (Logs)	461(2.6)	20(1.3)	
Maximum (Logs)	2950000(6.4)	695000(5.8)	

### Viral load based on sex and age

Before HAART initiation males had a mean rank viral load 59.9 compared to 49.5 among females but this was not significant (P value= 0.090). Following HAART mean rank of drop in viral load achieved by males was 55.3 and by females 55.8 (P value= 0.766). Similarly, there was no significant difference between the age and mean rank of viral load before HAART initiation (P value= 0.918) as well as age and mean rank of viral load after 12 months of HAART initiation (P value= 0.063) (Table 5).

**Table 5:** Viral load level based on sex and age. However, there was no significant difference detected between the groups

Variables	N	Mean Rank	Mann-Whitney Test/ Kruskal-Wallis Test
**Viral load before HAART initiation based on sex**			
Male	63	59.94	0.090
Female	47	49.54	
**Viral load at 12 months of treatment based on sex**			
Male	63	55.25	0.766
Female	47	55.84	
**Viral load before HAART initiation based on age**			
18-30 years	28	56.02	0.918
31-40 years	52	54.25	
> 40 years	30	57.18	
**Viral load at 12 months of treatment based on age**			
18-30 years	28	59.38	0.063
31-40 years	52	53.5	
> 40 years	30	55.35	

### CD4+ cell recovery and level of viremia following HAART with regard to a target of >200 cells/μl

CD4 Count and viral load levels were analyzed based on whether a patient achieved a CD4 count of ≥ 200 cells/μl at 12 months of HAART initiation. Despite HAART, 29 (26.4%) of study participants were still having CD4 count below 200 cells/μl compared to 81 (73.6%) participants who had a CD4 count of more than 200 cells/μl as CD4 count. As for viral load, 106 (96.4%) patients achieved a net decrease in viremia to 20 RNA copies/ml while 4 (3.6%) had an increase in their viremia (Table 6).

**Table 6:** CD4^+^ cell recovery and level of viremia following HAART with regard to a target of >200 cells/μl

CD4 cells after Treatment	Frequency	Percentage
CD4 count ≤ 200 cells/μl	29	26.4
CD4 count > 200 cells/μl	81	73.6
Viremia decrease to up to 20 RNA/ml	106	96.4
Increase in viremia	4	3.6

### Association between demographics and CD4 count level

Table 7 shows the relationship between demographic characteristics and CD4 count level after 12 months of HAART initiation. Respondents aged 18-30 years were significantly more likely to have less than 200 cells/μl CD4 count (46.4%) [OR=4.33; 95%CI: 1.29-14.59; P=0.018] than those respondents aged above 40 years (16.7%). However, there was no significant association (P<0.05) between sex and CD4 count level after 12 months of HAART initiation.

**Table 7:** Association between demographics and CD4 count level

Variables	Total, N	CD4 count ≤ 200 cells/μl, n(%)	CD4 count > 200 cells/μl, n(%)	OR (95% CI)	Chi square ^+^p value
**Sex**					
Male	63	17(27.0%)	46(73.0%)	1.10(0.46-2.55)	0.864
Female	47	12(25.5%)	35(74.5%)	1.00	
**Age in years**					
18-30	28	13(46.4%)	15(53.6%)	4.33(1.29-14.59)	**0.018^+^**
31-40	52	11(21.2%)	41(78.8%)	1.34(0.42-4.32)	0.622
> 40	30	5(16.7%)	25(83.3%)	1.00	

OR= Odds Ratio; CI= Confidence Interval;

+ Significant p value

## Discussion

The World Health Organization recommends a limit CD4 count of 350 cells/μl or clinical stage based decision for HAART initiation; particularly in countries where viral load cannot be easily determined [7, 17] and a CD4 count of less than 200 cells/μl at HAART initiation indicates a severe immunosuppression among HIV+ patients [18]. In the present study; CD4+ cell recovery was assessed among HIV+ patients who initiated HAART with a CD4 counts of less than 200 cells/μl. Comparison between CD4 count, viral load before HAART and after 12 months of HAART was done among HIV positive patients with severe immunosuppression.

A significant recovery of CD4 positive cells and a drop in viremia following 12 months of HAART among study participants were documented and this is in agreement with findings by [7] and a study by [6]. Low CD4 count and high viral load are indicators of HIV associated immunosuppression and this explains why HAART is initiated for people with CD4 count of less than 350 cells/μl and more particularly why HIV+ patients with CD4 count below 200 cells/μl are immediately put on HAART [19]. Though most of study participants achieved a net drop in viral load following 12 months of HAART; a number of patients did not get a drop in viremia and this can suggest a treatment failure that can be associated with factors like viral mutations, regimens or defaulting during HAART [20].

Some patients saw a decrease in their viral load after 12 months of treatment. However; this was not associated with a net recovery of CD4+ cells to reach more than 200 cells/μl; as following the 12 months of HAART considerable percentage (26.4%) of participants were still having CD4 count below 200 cells/μl compared to 73.6% of participants who achieved more than 200 cells/μl as CD4 count. This finding highlights the importance of viral load for the biological follow up of HIV+ patients both before and following HAART compared to CD4 count. Several studies have reported a poor prognosis prediction by CD4 count as it sometimes does not correlate with viral load in what is known as discordant response [21] and slowness in immune system recovery when HIV+ patients start HAART with severe immunosuppression have also been highlighted [22].

Poor immunologic response has been attributed to patient factors like age, the regimen as well as viral factors. However; this was not the case in the present study where young patients were likely to have a poor immune recovery compared to old patients [23]. The present study has again shown how important is to start HAART before a severe immunosuppression; for a better immune recovery. A difference in CD4+ cells recovery between a group of HIV+ patients with severe immunosuppression and a group with CD4 count >200 cells/μl at HAART; has also been reported at six months of HAART by [6]. During this study, there was no difference in CD4 count and viral load between males and females at both time points (P>0.005). This had also been documented by Annison & Dompreh (2013) at the Komfo Anokye Teaching Hospital, Ghana [24]. Younger participants were likely to have lower CD4 count but this was not associated with in increase in viremia and could be attributed to prognostic prediction discrepancies between viral load and CD4 count [6].

## Conclusion

We conclude that immune recovery is slow among HIV+ patients who start HAART with severe immunosuppression. Early HAART intervention is necessary for achieving effective CD4+ T cell responses and optimal immunological function in HIV+ patients.

### What is known about this topic

- Several researchers have focused their attention on CD4+ cells recovery among HIV+ patients following HAART in general.

### What this study adds

- This study has enriched the knowledge on how CD4+ cells recover among HIV+ patients when they initiate HAART after a severe immunosuppression;
- The study has again pointed out the importance of early HAART initiation among HIV+ patients particularly in the context of Rwanda where such data were scarce;
- The present study will also raise more interests among researchers on host cellular and molecular as well as viral factors behind this poor recovery in CD4+ cells.
